# Assessing strategies to target screening for advanced liver fibrosis among overweight and obese patients

**DOI:** 10.20517/mtod.2022.08

**Published:** 2022-07-18

**Authors:** Fernando Bril, Eddison Godinez Leiva, Romina Lomonaco, Sulav Shrestha, Srilaxmi Kalavalapalli, Meagan Gray, Kenneth Cusi

**Affiliations:** 1 Division of Endocrinology, Diabetes and Metabolism, Department of Medicine, University of Alabama at Birmingham, Birmingham, AL 35233, USA.; 2 Division of Endocrinology, Diabetes and Metabolism, Department of Medicine, University of Florida, Gainesville, FL 32608, USA.; 3 Division of Gastroenterology, Hepatology, and Nutrition, Department of Medicine, University of Alabama at Birmingham, Birmingham, AL 35233, USA.

**Keywords:** Nonalcoholic fatty liver disease, NAFLD, diagnosis, NASH, non-invasive

## Abstract

**Aim:**

The optimal screening strategy for advanced liver fibrosis in overweight and obese patients is unknown. The aim of this study is to compare the performance of different strategies to select patients at high risk of advanced liver fibrosis for screening using non-invasive tools.

**Methods:**

All patients underwent: liver ^1^H-MRS and percutaneous liver biopsy (in those with nonalcoholic fatty liver disease [NAFLD]). Unique selection strategies were compared to determine the best screening algorithm: (A) A “metabolic approach”: selecting patients based on HOMA-IR ≥ 3; (B) A “diabetes approach”: selecting only patients with type 2 diabetes; (C) An “imaging approach”: selecting patients with hepatic steatosis based on ^1^H-MRS; (D) A “liver biochemistry approach”: selecting patients with elevated ALT (i.e., ≥ 30 IU/L for males and ≥ 19 IU/L for females); and (E) Universal screening of overweight and obese patients. FIB-4 index, NAFLD fibrosis score, and APRI were applied as screening strategies.

**Results:**

A total of 275 patients were included in the study. Patients with advanced fibrosis (*n* = 29) were matched for age, gender, ethnicity, and BMI. Selecting patients by ALT elevation provided the most effective strategy, limiting the false positive rate while maintaining the sensitivity compared to universal screening. Selecting patients by any other strategy did not contribute to increasing the sensitivity of the approach and resulted in more false positive results.

**Conclusion:**

Universal screening of overweight/obese patients for advanced fibrosis with non-invasive tools is unwarranted, as selection strategies based on elevated ALT levels lead to the same sensitivity with a lower false positive rate (i.e., fewer patients that would require a liver biopsy or referral to hepatology).

## INTRODUCTION

Nonalcoholic fatty liver disease (NAFLD) has become a serious public health problem with an estimated prevalence of ~25% in the overall population^[1]^. Among overweight or obese patients, this prevalence is probably much higher in the range of 50%−60%^[2,3]^. Despite this high prevalence, there is still no established algorithm to screen for liver disease in these patients. A percutaneous liver biopsy remains the gold standard for the diagnosis of nonalcoholic steatohepatitis (NASH) and for determining the liver fibrosis stage^[4]^. Numerous non-invasive tools, including plasma biomarkers, imaging tests, and clinical scores combining demographic and biochemical data, have been proposed^[5,6]^. They have been widely used to predict the presence of NAFLD, NASH, and/or advanced fibrosis. Moreover, national and international guidelines have even endorsed some of these approaches^[4,7,8]^. However, a clear consensus regarding which patients would benefit the most from advanced fibrosis screening is lacking. Specifically, it is unclear whether all overweight or obese patients should be screened for hepatic fibrosis, or if screening should be reserved only for selected patients at high risk of liver fibrosis. In which case, the best strategy to select these high-risk patients is currently unknown. While different approaches have been proposed (i.e., selecting patients based on the presence of diabetes, presence of metabolic syndrome, elevated plasma aminotransferases, or presence of hepatic steatosis)^[8]^, no prior study has compared these approaches head-to-head. Therefore, the aim of this study is to compare different strategies to target screening for advanced liver fibrosis in overweight and obese patients.

## METHODS

### Research subjects

Patients included in this study were recruited from hepatology and endocrinology clinics at the University of Florida in Gainesville, FL and the University of Texas Health Science Center at San Antonio (UTHSCSA) in San Antonio, TX, as well as from the general population. Patients included in this manuscript were previously included in other manuscripts assessing the metabolic implications of NAFLD and NASH or assessing therapies for NASH^[9–11]^. Adult overweight or obese (BMI ≥ 25 kg/m^2^) patients were included in the study after exclusion of secondary liver diseases (hepatitis B or C, autoimmune hepatitis, hemochromatosis, Wilson’s disease, drug-induced hepatitis, *etc*.), significant alcohol consumption (≥ 30 gm/day for males and ≥ 20 gm/day for females), type 1 diabetes mellitus, or use of medications that can affect intrahepatic triglyceride content (i.e., vitamin E, pioglitazone, weight loss medications, amiodarone, glucocorticoids, methotrexate, tamoxifen, olanzapine, protease inhibitors). Patients were included regardless of the presence of type 2 diabetes (T2D). However, patients were excluded if they were currently prescribed SGLT-2 inhibitors, GLP-1 agonists, and/or pioglitazone. Moreover, all glucose- and lipid-lowering medications, as well as physical activity and diet, were required to be stable for at least three months prior to participation in the study. The study was approved by the institutional review boards at the University of Florida (UF) and the UTHSCSA, and written informed consent was obtained from each patient prior to their participation.

### Study design

In this cross-sectional study, patients underwent a liver ^1^H-MRS, a percutaneous liver biopsy (if diagnosed with NAFLD by imaging), and baseline measurements of all components of fibrosis-4 index (FIB-4), NAFLD fibrosis score (NFS) and AST to platelet ratio index (APRI). Patients with incomplete data were excluded from the study. Universal screening for advanced fibrosis in overweight and obese patients was compared against different strategies to select a high-risk subpopulation based on typical information available in the clinical setting. The different approaches aimed to target screening for advanced fibrosis were: (A) A “metabolic approach”: selecting only patients with elevated insulin resistance based on HOMA-IR ≥ 3; (B) A “diabetes approach”: selecting only patients with T2D; (C) A “imaging approach”: selecting patients with known hepatic steatosis based on ^1^H-MRS with intrahepatic triglyceride content ≥ 5.6%; (D) A “liver biochemistry approach”: selecting patients with elevated ALT (i.e., ≥ 30 IU/L for males and ≥ 19 IU/L for females). These were compared to a “universal screening approach”: all overweight and obese patients were screened. Head-to-head comparisons were performed among different diagnostic strategies to assess their accuracy in detecting patients with advanced fibrosis and the number of biopsies needed for that. For the purposes of this study, the assumption was that any patient with a positive screening would undergo a liver biopsy.

### Intrahepatic triglyceride content and histology

Intrahepatic triglyceride accumulation was measured by liver ^1^H-MRS as previously described^[12]^. The intrahepatic triglyceride content of ≥ 5.6% was considered diagnostic of NAFLD^[4,13]^. Patients without NAFLD were considered as not having advanced fibrosis for the purpose of this study. Percutaneous liver biopsies were performed with US guidance in those with NAFLD considered at high risk for progressive liver disease. All biopsies were blindly read by the same pathologist, who was unaware of the patient’s characteristics. Diagnosis of NASH was made following standard criteria^[14]^: the presence of zone 3 macrovesicular steatosis (any grade), hepatocellular ballooning (any degree), and lobular inflammatory infiltrate (any amount). Fibrosis was classified based on standard criteria^[15]^. Advanced fibrosis was defined as the presence of fibrosis stages 3 or 4 (F3 or F4). Clinically significant fibrosis (or moderate fibrosis) was defined as fibrosis stages 2 or higher (i.e., ≥ F2). NAFLD activity score (NAS) was calculated as the sum of the steatosis, inflammation, and ballooning grades in the liver biopsy. The mean length of liver biopsies was 1.6 cm (95%CI: 1.5–1.7 cm).

### Non-invasive scores used to diagnose advanced fibrosis

Based on simplicity and widespread availability, NFS, FIB-4, and APRI were used as non-invasive biomarkers for the prediction of advanced fibrosis. Their formulas are public domain and can be found elsewhere^[16]^.

### Statistical analysis

Data has been summarized as number (percentage) for categorical variables and mean ± SD for continuous variables. Sensitivity, specificity, and positive and negative predictive values for the different approaches were calculated using histology as the gold standard for the diagnosis of advanced fibrosis. A two-tailed value of *P* < 0.05 was considered to indicate statistical significance. Analyses were performed with Stata 11.1 (StataCorp LP, College Station, TX) and graphs with Prism 6.0 (GraphPad Software, Inc., La Jolla, CA).

## RESULTS

### Patients’ characteristics

A total of 275 overweight and obese patients were included in the study. The proportion of patients with NAFLD was 67% [Supplementary Table 1]. Patients’ characteristics have been summarized in Table 1, where patients were divided based on the presence or absence of advanced fibrosis in the liver biopsy. As can be observed, groups were well-matched for important clinical and anthropometric characteristics, such as age, gender, ethnicity, weight, and BMI. Patients with advanced fibrosis had a higher prevalence of type 2 diabetes and a consequent higher fasting plasma glucose and hemoglobin A1c. As expected, patients with advanced fibrosis also showed higher levels of plasma AST and ALT; however, AST/ALT ratio was not different compared to patients without advanced fibrosis (0.92 ± 0.27 *vs*. 0.88 ± 0.31, *P* = 0.54). All histological parameters were also worse in patients with advanced fibrosis, except for steatosis, which was technically not significantly different between both groups (*P* = 0.06). However, intrahepatic triglyceride content based on liver ^1^H-MRS was significantly higher in patients with advanced fibrosis.

### Performance of the different strategies to identify patients with advanced fibrosis

The performance of different strategies to select patients at risk of advanced fibrosis was compared by applying three different, widely available, non-invasive clinical scores as the screening approach: FIB-4 index [Table 2], NAFLD fibrosis score [Table 3], and APRI [Table 4].

#### FIB-4 index

All strategies using FIB-4 index, except the ones based on measuring insulin resistance or selecting for the presence of diabetes, led to the identification of 22 of the 29 patients with advanced fibrosis (i.e., similar sensitivity of 76% across the different strategies) [Table 2]. The main differences among these strategies are related to the rate of false positives and, therefore, the potential number of biopsies that would be needed, assuming that all patients with a positive screening would be advised to undergo a percutaneous liver biopsy. Universal screening did not help to identify more patients with advanced fibrosis compared to more restrictive approaches, like the imaging approach or the liver biochemistry approach. Based on a universal approach, 41 biopsies would be needed for every 100 patients screened, which means ~5.18 biopsies performed per each patient identified with advanced fibrosis. Identification of patients based on elevated ALT was the strategy that allowed for the lowest false positive rate while maintaining sensitivity. This strategy would result in the need for 25 biopsies for every 100 patients screened, reducing to only ~3.18 biopsies needed to identify one patient with advanced fibrosis. Results for the imaging approach were very similar to the approach using elevated ALT [Table 2]. The metabolic and diabetes approaches required a higher number of biopsies compared to the liver biochemistry approach and resulted in a lower sensitivity to detect advanced fibrosis.

#### NAFLD fibrosis score

Applying NAFLD fibrosis score allowed for higher sensitivities compared to FIB-4 index, but significantly higher false positive rates [Table 3]. Universal screening of overweight and obese patients resulted in 229 out 275 patients with a positive screening (i.e., need for 85 biopsies for every 100 patients screened or ~8.18 biopsies to identify one patient with advanced fibrosis). The need for biopsies was reduced by selecting patients based on insulin resistance (42 biopsies per 100 screened patients), presence of diabetes (56 biopsies per 100 screened patients), presence of NAFLD (54 biopsies per 100 screened patients), or abnormal ALT (50 biopsies per 100 screened patients). The metabolic and liver biochemistry approaches were the ones with the lower number of biopsies needed to identify one patient with advanced fibrosis (4.64 and 4.85, respectively). However, the liver biochemistry approach had a higher sensitivity (97% *vs*. 86%). Of note, using the NAFLD fibrosis score resulted in the need for significantly more biopsies compared to the FIB-4 index, regardless of the population selection strategy.

#### APRI

Using APRI to screen for advanced fibrosis in overweight and obese patients resulted in sensitivities similar to FIB-4 index, but overall lower false positive rates [Table 4]. A universal screening approach with APRI allowed to identify 22 out of 29 patients with advanced fibrosis, and it resulted in the need for 67 biopsies among 275 patients (24 out of 100 patients screened; 3.05 biopsies per patient identified with advanced fibrosis). No significant differences were observed if patients were preselected based on the presence of NAFLD (23 biopsies for every 100 patients screened) or abnormal ALT (24 biopsies out of 100 patients screened). The number needed for biopsies to diagnose one patient with advanced fibrosis with these approaches was 2.81 and 2.95, respectively. The use of elevated HOMA-IR for identification of patients allowed to reduce the number of biopsies to 18 for every 100 patients screened, but sensitivity dropped from 76% to 69% (i.e., would result in missing 0.7 patients with advanced fibrosis for every 100 patients screened).

### Using AST, AST/ALT ratio, or other ALT cut-off levels to select patients

As the development of fibrosis in NAFLD has been associated with increasing plasma AST and AST/ALT ratio, we also assessed if selecting patients by these would be helpful for targeting screening. However, plasma AST and AST/ALT ratio performed very similarly to plasma ALT, without significant changes in the number needed to biopsy or overall sensitivity (data not shown). Using ALT to select patients with a more conservative cut-off point of > 40 IU/L led to a reduction in the need for biopsies per 100 patients screened with FIB-4 index (i.e., 19 compared to 25 with the cut-off points of 19 and 30 IU/L depending on sex), but also a significant drop in sensitivity (i.e., from 76% to 66%). Similar changes were observed if the higher ALT threshold was used with NAFLD fibrosis score or APRI as the screening tool.

### Screening for clinically significant fibrosis

In Table 5, we have provided information regarding the use of these different strategies for the identification of patients with clinically significant fibrosis (*n* = 49). As expected, these approaches were less sensitive to detecting patients with clinically significant fibrosis but had lower false positive rates. Universal screening using FIB-4 allowed to identify 76% of patients with clinically significant fibrosis but required 3.08 biopsies per patient identified (41 biopsies per 100 patients screened). This could be reduced to ~2 biopsies needed to identify one patient with clinically significant fibrosis by targeting patients with elevated ALT or presence of NAFLD (with sensitivity only minimally reduced to 73%). Using the NFS resulted in overall higher sensitivities, but with higher false positive rates. Similar to what happened with the FIB-4 index, targeting patients with elevated plasma ALT allowed to diminish the number of needed biopsies without significantly affecting the sensitivity. On average, this approach required 2.96 biopsies for every patient identified with clinically significant fibrosis with an overall sensitivity of 94%. Finally, screening with APRI resulted in a similar sensitivity than with the FIB-4 index, but required a lower number of biopsies. Moreover, universal screening with APRI did not result in a significant increase in the number of biopsies needed compared to other selection strategies like elevated ALT or the presence of NAFLD.

### Other sensitivity analyses

We also repeated the analyses limiting the cohort to obese patients (BMI ≥ 30kg/m^2^), without observing significant differences. Finally, we repeated the analyses, excluding 92 patients that did not have a liver biopsy. These patients did not have NAFLD based on liver ^1^H-MRS and had normal plasma aminotransferases and therefore were not offered a percutaneous liver biopsy as this would be considered unethical. Because all these patients had a negative ^1^H-MRS and normal plasma aminotransferases, they were assumed not to have significant fibrosis. Therefore, excluding these patients did not affect the sensitivity of the different strategies. No significant differences were observed when excluding these patients.

## DISCUSSION

The best strategy to identify patients that would benefit from advanced liver fibrosis screening among overweight and obese patients is currently unknown. In the current work, we showed that using non-invasive strategies (i.e., FIB-4 index, NFS, or APRI), universal screening of overweight and obese patients is not justified. If broadly applied, this would lead to a significantly higher number of false positive results, compared to more restrictive strategies. Specifically, targeting screening to only patients with evidence of NAFLD or those with elevated ALT led to the most effective screening strategies, decreasing the need for liver biopsies while maintaining the same sensitivity. Strategies focused on selecting patients based on the presence of insulin resistance by HOMA-IR or the presence of diabetes resulted in lower sensitivities.

Recently, the American Gastroenterological Association (AGA), in collaboration with members of other societies, published a NAFLD/NASH Clinical Care Pathway^[8]^. In this work, the authors identified three groups of patients at greatest risk of NAFLD/NASH-related fibrosis: 1) patients with T2D, 2) patients with two or more metabolic risk factors, and 3) patients with incidental findings of hepatic steatosis or elevated aminotransferases. The authors recommended that these groups of patients would benefit the most from screening for significant liver fibrosis with noninvasive testing. However, these recommendations were based on experts’ opinions, and no prior study has formally assessed the performance of different patient selection strategies for the screening of liver fibrosis. It is well known, however, that patients can have significant liver disease in the absence of plasma aminotransferase elevations^[17]^. Therefore, it seems intuitive including other ‘at risk’ groups in the guidelines, such as patients with diabetes and those with metabolic risk factors. However, these and other guidelines^[4,8,18]^ recommend initiating the screening for advanced liver fibrosis with non-invasive tests, which rely heavily on plasma aminotransferase elevations, so patients with normal plasma aminotransferases may still go undiagnosed with these tools. Therefore, adding more ‘at risk’ groups may not provide an advantage over just selecting patients based on elevated plasma aminotransferases. Indeed, our results suggest that an approach that only selects patients based on elevated aminotransferases allows for the maximal sensitivity possible (equal to universal screening) with the least number of patients with positive results requiring further testing. The addition of patients based on other criteria (i.e., HOMA-IR, diabetes, presence of NAFLD) did not increase the detection of patients and only resulted in a higher rate of false positives.

For the purposes of this study, the assumption was that patients with a positive screening would undergo a liver biopsy. However, we are aware that this is not what usually occurs in clinical practice, as patients frequently undergo further testing before getting a biopsy. Regardless, the results from our study are still valid, as it helps to delineate the best selection strategy to target the second step of the diagnostic algorithm. As vibration-controlled transient elastography (VCTE) or Fibroscan^®^ becomes more widely available, it is likely that it will be increasingly used to diagnose advanced fibrosis in NAFLD^[19]^. Moreover, it has been proposed as a second step in the diagnostic algorithm by recent guidelines^[8]^. However, because it is still not widely available, especially in developing countries, it was important to establish the most effective way to screen for advanced fibrosis using only simple, cheap, and widely available tools.

Screening by means of APRI, instead of the FIB-4 index, resulted in significant changes in the performance of the different selection strategies. Indeed, universal screening with APRI was as effective as other selection strategies (i.e., presence of NAFLD or elevated ALT), reducing the number of needed biopsies while keeping the sensitivity stable. This suggests that APRI, unlike FIB-4 index, may be used in an ‘untargeted’ fashion on overweight and obese patients without the need to pre-identify specific subgroups. Nevertheless, identifying patients with the presence of NAFLD or elevated ALT did not affect the sensitivity of the approach and, if anything, allowed to reduce the number of biopsies by ~1 per 100 patients screened. NFS allowed to increase the detection of patients with advanced fibrosis (higher sensitivity), but this occurred at the expense of a significantly higher false positive rate. Universal screening of overweight and obese patients with NFS is unlikely to be cost-effective as it leads to an overwhelming number of patients with a positive test. Once again, the selection of patients by elevated ALT was the most effective approach, allowing for the lowest number of biopsies while maintaining sensitivity. Because the NFS is calculated based on BMI and the presence of impaired fasting glucose or diabetes, high false positive rates may be related to the characteristics of our cohort. Patients were selected based on increased BMI (≥ 25 kg/m^2^) and 91% of patients had prediabetes or diabetes, likely related to referral bias.

Overall and liver-related mortality in patients with NAFLD appears to be related to the fibrosis stage, and it significantly increases once the fibrosis stage is 2 or higher^[20]^. Therefore, identifying patients with clinically significant fibrosis (stages ≥ 2) is essential to provide appropriate counseling and offer potential treatments. While these approaches were not particularly sensitive to detecting patients with clinically significant fibrosis, using FIB-4 index or APRI in patients with elevated ALT allowed to limit biopsies to 24 or 25, respectively, for every 100 patients screened. Moreover, less than two biopsies were needed (1.94 and 1.81, respectively) to identify one patient with clinically significant fibrosis (i.e., > 50% of patients undergoing a liver biopsy had clinically significant fibrosis).

The study has some limitations, many of which are inherent to the nature of the study. As performing a liver biopsy in patients without NAFLD would be unethical, these patients were assumed to not have significant fibrosis. However, as liver fibrosis progresses, the amount of intrahepatic triglyceride can decrease, which can lead to negative imaging for hepatic steatosis. The other important limitation is that our cohort was likely enriched with patients with metabolic abnormalities due to referral and selection bias. Finally, this is a relatively small sample size. Therefore, confirmation in larger cohorts is important before we can extrapolate and generalize our results.

In summary, the current study suggests that when using non-invasive tests to screen for advanced fibrosis (FIB-4, NFS, or APRI) in overweight and obese patients, targeted screening of patients with elevated ALT provides the most cost-effective approach, reducing the number of needed biopsies while maintaining the sensitivity. Universal screening of these patients resulted in unnecessary false positive results without increasing the number of patients identified with advanced fibrosis. Selecting patients based on insulin resistance or the presence of diabetes did not provide any further advantage compared to elevated ALT alone. If confirmed in larger cohorts of patients, our results provide a simple strategy to identify patients to screen for advanced fibrosis among overweight and obese patients, reducing the number of liver biopsies needed to identify these patients.

## Supplementary Material

Supplementary material

## Figures and Tables

**Table 1.** Patients’ characteristics based on the presence of advanced fibrosis (F3 or F4)

	Patients without advanced fibrosis (*n* = 246)	Patients with advanced fibrosis (*n* = 29)	*P* value
**Age, years**	54 ± 11	54 ± 9	0.99
**Gender, male/female %**	74%/26%	72%/28%	0.86
**Ethnicity**			0.54
**Caucasian, %**	46%	45%	
**Hispanic, %**	37%	48%	
**African-American, %**	15%	7%	
**Other, %**	2%	-	
**Weight, kg**	99 ± 17	101 ± 16	0.59
**Body mass index, kg/m** ^**2**^	33.8 ± 4.9	35.1 ± 5.3	0.20
**Presence of diabetes, %**	59%	86%	0.004
**Diabetes medications**			
**Metformin, %**	41%	72%	0.001
**Sulfonylureas, %**	22%	48%	0.003
**Insulin, %**	12%	34%	0.003
**Statin use, %**	58%	59%	0.92
**Blood pressure medication use, %**	69%	83%	0.12
**A1c, %**	6.4 ± 1.1	7.4 ± 1.3	< 0.001
**Fasting plasma glucose, mg/ml**	125 ± 34	147 ± 41	0.002
**Intrahepatic triglyceride content, %**	11 ± 10	15 ± 7	0.037
**Aspartate aminotransferase, U/L**	32 ± 19	63 ± 37	< 0.001
**Alanine aminotransferase, U/L**	43 ± 34	73 ± 44	0.005
**NAFLD activity score**	3.1 ± 1.5	5.0 ± 1.3	< 0.001
**Steatosis grade**	1.5 ± 0.8	1.8 ± 0.6	0.06
**Patients with grade 0–1**	55%	28%	
**Patients with grade 2**	31%	66%	
**Patients with grade 3**	13%	7%	
**Inflammation grade**	1.1 ± 0.6	1.7 ± 0.5	< 0.001
**Patients with grade 0**	11%	0%	
**Patients with grade 1**	71%	34%	
**Patients with grade 2–3**	18%	66%	
**Ballooning grade**	0.5 ± 0.6	1.6 ± 0.6	< 0.001
**Patients with grade 0**	56%	7%	
**Patients with grade 1**	38%	31%	
**Patients with grade 2**	6%	62%	
**Fibrosis stage**	0.6 ± 0.7	3.1 ± 0.3	< 0.001
**Patients with stage 0**	52%	0%	
**Patients with stage 1**	37%	0%	
**Patients with stage 2**	11%	0%	
**Patients with stage 3–4**	0%	100%	

**Table 2.** Performance of different strategies to identify patients with advanced fibrosis (F3 or F4) based on the FIB-4 index

Selection Strategy	Number of patients selected	Patients with FIB-4 ≥ 1.30	Patients identified with advanced fibrosis	Number of biopsies per patient identified with advanced fibrosis	Patients that would need a biopsy per 100 patients	Sensitivity of the approach (%)	False positive rate (%)
**Metabolic approach** HOMA-IR ≥ 3	129	67	20	3.35	24	69	19
**Diabetes approach** Patients with T2D	169	87	20	4.35	32	69	27
**Imaging approach** NAFLD by ^1^H-MRS	183	74	22	3.36	27	76	21
**Liver biochemistry approach** ALT ≥ 30 IU/L in males and ≥ 19 IU/L in females	168	70	22	3.18	25	76	20
**Universal screening** All patients	275	114	22	5.18	41	76	37

**Table 3.** Performance of different strategies to identify patients with advanced fibrosis (F3 or F4) based on NFS

Screening strategy	Number of patients selected	Patients with NFS ≥ −1.455	Patients identified with advanced fibrosis	Number of biopsies per patient identified with advanced fibrosis	Patients that would need a biopsy per 100 patients	Sensitivity of the approach (%)	False positive rate (%)
**Metabolic approach** HOMA-IR ≥ 3	129	116	25	4.64	42	86	38
**Diabetes approach** Patients with T2D	169	151	24	6.29	56	83	53
**Imaging approach** NAFLD by ^1^H-MRS	183	147	27	5.44	54	93	50
**Liver biochemistry approach** ALT ≥ 30 IU/L in males and ≥ 19 IU/L in females	168	136	28	4.85	50	97	42
**Universal screening** All patients	275	229	28	8.18	85	97	84

**Table 4.** Performance of different strategies to identify patients with advanced fibrosis (F3 or F4) based on APRI

Screening strategy	Number of patients selected	Patients with APRI ≥ 0.50	Patients identified with advanced fibrosis	Number of biopsies per patient identified with advanced fibrosis	Patients that would need a biopsy per 100 patients	Sensitivity of the approach (%)	False positive rate (%)
**Metabolic approach** HOMA-IR ≥ 3	129	50	20	2.50	18	69	12
**Diabetes approach** Patients with T2D	169	43	19	2.26	16	66	10
**Imaging approach** NAFLD by ^1^H-MRS	183	62	22	2.81	23	76	16
**Liver biochemistry approach** ALT ≥ 30 IU/L in males and ≥ 19 IU/L in females	168	65	22	2.95	24	76	17
**Universal screening** All patients	275	67	22	3.05	24	76	18

**Table 5.** Performance of different strategies to identify patients with clinically significant fibrosis (≥ F2) based on FIB-4 index, NFS, and APRI

Selection strategy	Number of patients selected	Patients with a positive test	Patients identified with clinically significant fibrosis	Number of biopsies per patient identified with clinically significant fibrosis	Patients that would need a biopsy per 100 screened	Sensitivity (%)	False positive rate (%)
**FIB-4 index** ≥ **1.30**							
**Metabolic approach** HOMA-IR ≥ 3	129	67	32	2.09	24	65	15
**Diabetes approach** Patients with T2D	169	87	31	2.80	32	63	25
**Imaging approach** NAFLD by ^1^H-MRS	183	74	36	2.06	27	73	17
**Liver biochemistry approach** ALT ≥ 30 IU/L in males and ≥ 19 IU/L in females	168	70	36	1.94	25	73	15
**Universal screening** All patients	275	114	37	3.08	41	76	34
**NAFLD fibrosis score** ≥ **−1.455**							
**Metabolic approach** HOMA-IR ≥ 3	129	116	40	2.90	42	82	35
**Diabetes approach** Patients with T2D	169	151	37	4.08	56	76	52
**Imaging approach** NAFLD by ^1^H-MRS	183	147	45	3.27	54	92	46
**Liver biochemistry approach** ALT ≥ 30 IU/L in males and ≥ 19 IU/L in females	168	136	46	2.96	50	94	41
**Universal screening** All patients	275	229	47	4.87	85	96	83
**APRI** ≥ **0.50**							
**Metabolic approach** HOMA-IR ≥ 3	129	50	33	1.67	18	67	8
**Diabetes approach** Patients with T2D	169	43	28	1.54	16	57	7
**Imaging approach** NAFLD by ^1^H-MRS	183	62	35	1.77	23	71	12
**Liver biochemistry approach** ALT ≥ 30 IU/L in males and ≥ 19 IU/L in females	168	65	36	1.81	24	73	13
**Universal screening** All patients	275	67	36	1.86	24	73	14

## Data Availability

Data available upon request to corresponding authors.
