# Supplementary material for: Assessing strategies to target screening for advanced liver fibrosis among overweight and obese patients
Source: Metab Target Organ Damage. Author manuscript; Available in PMC 2022 Aug 24. (PMC9400455; doi:10.20517/mtod.2022.08)
Supplement: Supplementary material [file NIHMS1824412-supplement-Supplementary_material.pdf]

Supplemental Table 1. Patients' characteristics based on the presence of NAFLD

	**Patients without NAFLD (n = 92)**	**Patients with NAFLD (n = 183)**	***P* value**
**Age, years**	58 ± 11	52 ± 11	< 0.001
**Gender, male/female %**	73%/27%	74%/26%	0.79
**Ethnicity**			< 0.001
**Caucasian, %**	50%	44%	
**Hispanic, %**	23%	46%	
**African-American, %**	27%	8%	
**Other, %**	0%	2%	
**Weight, kg**	98 ± 18	100 ± 16	0.30
**Body mass index, kg/m^2**	33.2 ± 5.3	34.3 ± 4.8	0.08
**Presence of diabetes, %**	64%	60%	0.52
**Diabetes medications**			
**Metformin, %**	49%	43%	0.34
**Sulfonylureas, %**	26%	25%	0.82
**Insulin, %**	15%	14%	0.73
**Statin use, %**	60%	57%	0.67
**Blood pressure medication use, %**	74%	68%	0.31
**A1c, %**	6.5 ± 1.2	6.5 ± 1.1	0.72
**Fasting plasma glucose, mg/ml**	125 ± 39	128 ± 34	0.49
**Intrahepatic triglyceride content, %**	3 ± 1	16 ± 9	< 0.001
**Aspartate aminotransferase, U/L**	23 ± 12	41 ± 26	< 0.001
**Alanine aminotransferase, U/L**	23 ± 18	58 ± 37	< 0.001
